# Prevalence and correlates of fatigue in amyotrophic lateral sclerosis: A systematic review and meta-analysis

**DOI:** 10.1007/s10072-023-07119-7

**Published:** 2023-10-14

**Authors:** Abdullah Ashraf Hamad, Basma Ehab Amer, Nagham Bushara Abbas, Asmaa Zakria Alnajjar, Mostafa Meshref

**Affiliations:** 1 Faculty of Medicine, Menoufia University, Menoufia, Egypt; 2 Faculty of Medicine, Benha University, Benha, Egypt; 3 Faculty of Medicine, Zagazig University, Zagazig, Egypt; 4 Faculty of Medicine, Al-Azhar University, Gaza, Palestine; 5 Medical Research Group of Egypt, Negida Academy, Arlington, MA USA; 6 Department of Neurology, Faculty of Medicine, Al-Azhar University, Cairo, Egypt

**Keywords:** Amyotrophic lateral sclerosis, ALS, Motor neuron disease, Fatigue, Non-motor symptoms, Meta-analysis

## Abstract

**Objectives:**

This systematic review and meta-analysis aimed to determine the frequency and correlates of fatigue in patients with amyotrophic lateral sclerosis (ALS).

**Methods:**

Three databases were searched up to 2nd May 2023 to identify studies reporting fatigue frequency in ALS. Studies included had to identify ALS patients through one of ALS diagnostic criteria and measure fatigue by a validated tool with a specific cut-off value. Meta-analysis was conducted using RStudio's "meta" package with a random-effects model. Subgroup analyses and meta-regression explored the relationship between fatigue frequency in ALS and different covariates.

**Results:**

Eleven studies, compromising 1072 patients, met the inclusion criteria and were included in our analysis. The pooled frequency of fatigue across all studies was 48% (95% CI = 40% to 57%). Our subgroup analysis based on the ALSFRS-R revealed a higher frequency of fatigue in studies with lower scores (< 30) compared to those with higher scores (≥ 30), with a pooled frequency of 62% (95% CI = 43% to 79%) and 43% (95% CI = 37% to 49%), respectively. Also, the meta-regression analysis showed a significant negative association between fatigue and ALSFRS-R mean (*P* = 0.02). The included studies reported an association between fatigue and lower functional status and poorer quality of life in patients with ALS.

**Conclusion:**

Our findings suggest that fatigue is prevalent in almost half of ALS patients and is associated with lower functional status and poorer quality of life, highlighting the importance of assessing and managing fatigue in ALS patients.

## Introduction

Amyotrophic lateral sclerosis (ALS), also known as Lou Gehrig's disease, is a progressive neurodegenerative disease that affects upper and lower motor neurons and is ultimately fatal [1]. Most cases of ALS are sporadic with unknown causes and about 10% are of familial origin [2]. According to the most recent epidemiological studies, the prevalence of ALS is 6.22 and 5.20 per 100,000 persons in Europe and North America, respectively [3]. ALS is characterized by progressive muscle weakness and wasting, leading to atrophy, loss of function, and ultimately respiratory failure over time [1, 4]. In most cases, the time between the disease onset and dependence on ventilatory support ranges from two to four years [1]. The progression symptoms of ALS include motor changes associated with the loss of strength, balance and coordination, leading to limitation in activities and self-care [4].

In addition to the motor impairment, ALS patients could experience other non-motor manifestations which can affect the patient's well-being such as pain, apathy, depression, and fatigue [5–8]. Fatigue is one of the common non-motor symptoms reported by ALS patients, and its impact on the functional status and well-being of patients has been increasingly recognized [7, 9]. Fatigue in ALS has been defined as "reversible motor weakness and whole‐body tiredness that was predominantly brought on by muscular exertion and was partially relieved by rest” [10]. The etiology of fatigue in ALS is not well understood, however it appears to involve multiple factors. For example, cardiorespiratory deconditioning, muscle changes resulting from disuse, and psychological factors are of the proposed mechanisms [11, 12]. Fatigue in ALS is commonly experienced in two distinct ways: general fatigue, characterized by a sensation of weariness throughout the entire body, and physical fatigue, which is associated with muscle use and reversible motor weakness [10, 11]. In addition to ALS, studies on other neurological diseases, such as multiple sclerosis and Parkinson's disease, have reported high prevalence of fatigue and its related impacts [13, 14].

Despite the growing interest in this topic, there is still a lack of consensus on the prevalence, severity, and correlates of fatigue in ALS patients, which may have led to a limited understanding of how to effectively manage fatigue in ALS [11]. Therefore, we conducted this systematic review and meta-analysis to pool the available evidence regarding the frequency, correlates, and clinical implications of fatigue in patients with ALS.

## Methods

This systematic review was prepared in accordance with the Preferred Reporting Items for Systematic Reviews and Meta-Analyses (PRISMA) guidelines [15].

### Search strategy and screening

We conducted a comprehensive search using PubMed, Web of Science, and Scopus from inception to May 2, 2023, using the following search terms: ("Motor Neuron Disease" OR "Motor System Disease" OR "Gehrig Disease" OR "Lou Gehrig's Disease" OR "Lou Gehrig" OR "Charcot Disease" OR "Amyotrophic Lateral Sclerosis" OR "Lateral Sclerosis" OR "Guam Disease" OR “ALS”) AND (“fatigue” OR “tired” OR “lethargy” OR “lethargic” OR “lassitude”). Without removing duplicates, two authors independently screened the titles and abstracts of the records against the eligibility criteria [16]. Subsequently, a third author retrieved and reviewed the full texts of the remaining studies to determine their inclusion. Any discrepancies were resolved by consensus. We used the Rayyan web tool for the screening process [17].

### Eligibility criteria

Our review included studies meeting the following criteria: a) reporting the frequency of fatigue in ALS patients through cross-sectional or longitudinal studies; b) defining participants as ALS patients according to one of the ALS diagnostic criteria (such as El Escorial or Awaji criteria); c) measuring fatigue using a validated tool with a cut-off value that indicates the presence of fatigue. Studies were excluded if they met any of the following criteria: a) participants were identified through self-reporting of ALS presence, or the study did not report how ALS patients were identified; b) fatigue prevalence was not reported or was identified through complaints.

### Outcomes and quality assessment

Our primary outcome was to estimate the frequency of fatigue in ALS patients through validated measures. Also, we aimed to identify the associations of fatigue in ALS patients. To assess the quality of the methods used to estimate fatigue prevalence in the included studies, we followed the Joanna Briggs Institute (JBI) critical appraisal checklist for prevalence studies [18]. It is important to note that this quality assessment tool only evaluates the quality of the fatigue prevalence method, and not the overall quality of the entire study, as estimating fatigue prevalence may not be the primary outcome of the study.

### Data extraction

The data were extracted independently by two authors using an online data extraction form, which included the following information: a) characteristics of the study (such as study design, setting, sample size, and ALS diagnosis criteria); b) characteristics of the participants in each study, including age, sex, symptom duration, and Amyotrophic Lateral Sclerosis Functional Rating Scale- Revised (ALSFRS-R); c) study outcomes, including fatigue prevalence, fatigue measuring tool, and fatigue associations; and d) Risk of bias domains. For longitudinal studies, we considered the baseline data.

### Data analysis

We conducted our analysis using the R (v.4.3.0) programming language and the “meta” package of RStudio software for Windows [19]. The “metaprop” function was used to transform the number of patients with fatigue and the total sample size in each included study into a pooled meta-analysis of proportions. Quantitative synthesis for the frequency of fatigue in patients with ALS was performed using the random-effects model and the inverse variance method. We relied on the chi-square *P* value and the *I*^*2*^ test to assess the heterogeneity among the included studies. A chi-square *P* value of less than 0.1 and *I*^*2*^ values of ≥ 50% indicated high heterogeneity. We conducted a subgroup analysis utilizing different fatigue measuring tools, namely the Fatigue Severity Scale (FSS), Checklist Individual Strength (CIS), and ALS Specific Quality of Life-Revised (ALSSQoL). For studies reporting their results using both FSS and CIS tools, we conducted our analysis using their data based on FSS tool. We also performed a sensitivity analysis using their data based on the CIS tool to ensure consistency between both cases. Another subgroup analysis was performed based on the mean ALSFRS-R (i.e., ALSFRS-R ≥ 30 versus ALSFRS-R < 30). Meta-regression was conducted to explore the association between frequency of fatigue in ALS and continuous variables, such as the sample size, publication year, time from disease onset, mean ALSFRS-R and the number of males. Visual inspection of the funnel plot and Egger’s test were used to explore publication bias across the included studies [20].

## Results

### Characteristics of individual studies

**Fig. 1:**
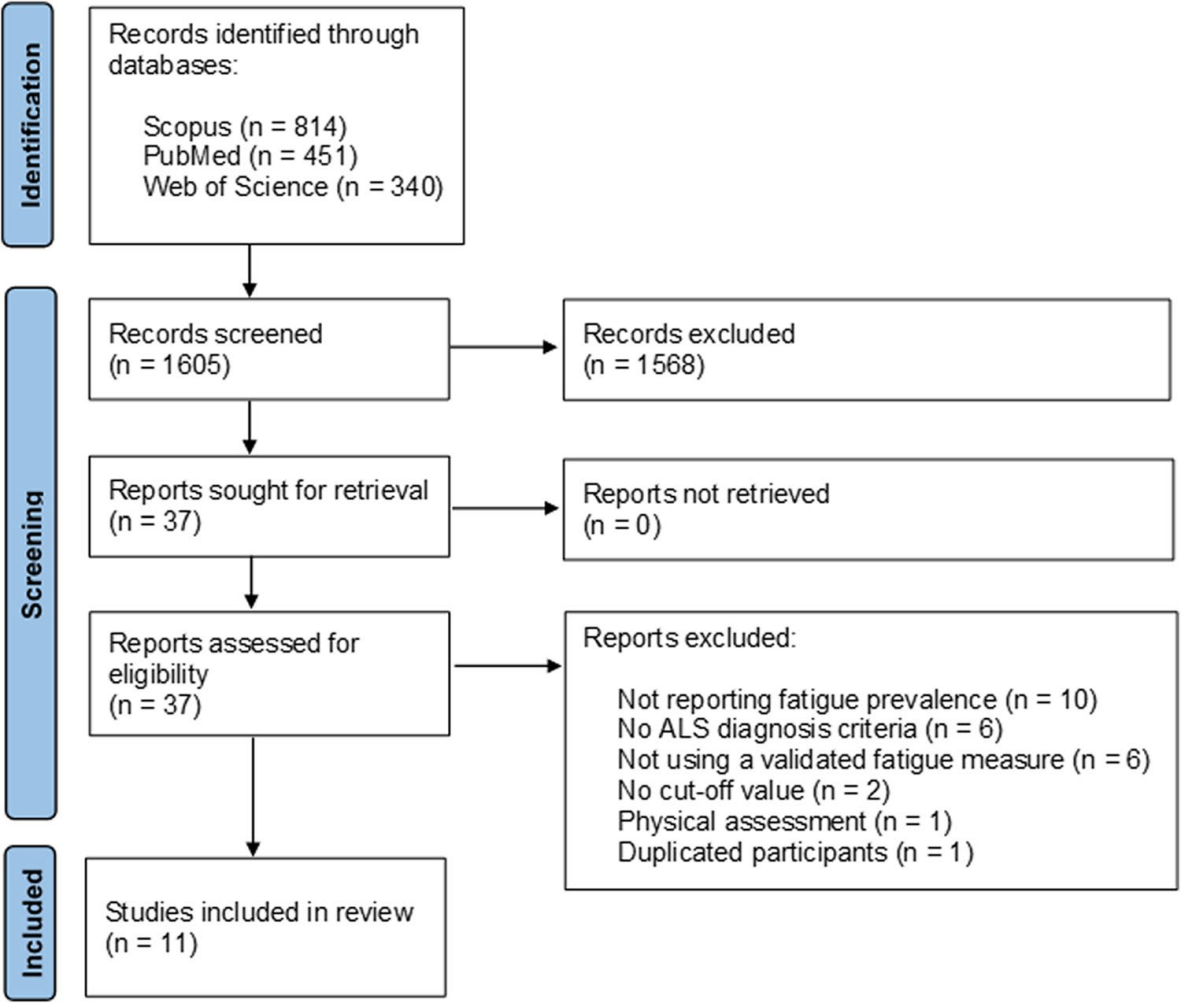
The PRISMA flow diagram

Our research yielded a total of 1605 citations. After screening the titles and abstracts, we identified 37 studies that were assessed against the eligibility criteria (Fig. 1). Of which, 10 studies did not report the prevalence of fatigue. Among the studies that reported fatigue prevalence, six studies did not provide criteria for defining ALS patients [21–26], six studies relied on patient self-report of fatigue presence without using a validated measure [27–32], two studies did not establish a specific cut-off value [12, 33], one study used a physiological measurement [34], and one study had the same participants of an included study [35]. Finally, 11 studies with 1072 ALS patients met the inclusion criteria and were included in this review [7, 9, 36–44]. These studies consisted of eight cross-sectional studies and three longitudinal studies, with sample sizes ranging from 51 to 223. Nine of the included studies used El Escorial criteria to define ALS patients. A summary of the included studies and their participants can be found in Table 1, while Table 2 presents the quality assessment of the studies.

**Table 1:** Characteristics of the included studies and their participants

Study ID	Study design	Country	ALS sample size	Age, mean (SD)	Male, n (%)	Time from disease onset in months, mean (SD)	Diagnosis criteria	ALSFRS-R, mean (SD)	Frequency of fatigue (%)	Fatigue measuring tool and categorization
Alencar 2022 a [7]	cross-sectional	Brazil	55	56.9 (11.2)	31 (56.4)	23.4 (15.8)	Awaji	29.5 (10.8)	45.5	FSS, a score ≥ 36 indicates the presence of fatigue
Alencar 2022 b [37]	cross-sectional	Brazil	65	56.6 (11.0)	40 (61.5)	75.6 (54.0)	Awaji	30.0 (10.9)	44.6	FSS, a score ≥ 36 indicates the presence of fatigue
An 2022 a [38]	cross-sectional	China	86	52.6 (10.9)	50 (58.1)	27.0 (16.8)	El Escorial	37.0 (5.3)	32.6	FSS, a score ≥ 36 indicates the presence of fatigue
An 2022 b [39]	cross-sectional	China	175	54.5 (3.0)	105 (60.0)	11.3 (1.7)	El Escorial	39.5 (1.5)	32.6	FSS, a score ≥ 36 indicates the presence of fatigue
Lococo 2012 [9]	cross-sectional	Italy	91	61.3 (10.1)	55 (60.4)	24.9 (15.4)	El Escorial	33.9 (8.8)	52.8	FSS, a score ≥ 36 indicates the presence of fatigue
McElhiney 2009 [40]	longitudinal	USA	223	61.0 (12.0)	98 (56.0)	-	El Escorial	33.0 (8.0)	44.0	FSS, a score > 40 indicates the presence of fatigue
Panitz 2015 [41]	longitudinal	Germany	51	57.9 (12.3)	24 (47.1)	15.8 (12.7)	El Escorial	38.7 (6.8)	59.0	FSS, a score > 36 indicates the presence of fatigue
									40.0	CIS, a score > 35 indicates the presence of fatigue
Raheja 2016 [42]	longitudinal	USA	82	53.0 (10.3)	46 (56.0)	-	El Escorial	38.5 (6.0)	42.7	ALSSQoL fatigue subscale, a score ≥ 4 indicates the presence of fatigue
Sandstedt 2016 [43]	cross-sectional	Sweden	51	61.0 (12.0)	28 (56.0)	37.3 (29.9)	El Escorial	28.7 (12.9)	61.0	CIS, a score ≥ 35 indicates the presence of fatigue
Vangroenestijn 2017 [44]	cross-sectional	Netherland	72	59.9 (10.6)	50 (69.4)	17.1 (12.2)	El Escorial	42.1 (3.7)	42.0	CIS, a score ≥ 35 indicates the presence of fatigue
Vogt 2020 [36]	cross-sectional	Germany	121	62.3 (9.3)	72 (59.5)	27.0 (34.0)	El Escorial	27.8 (9.5)	76.7	FSS, a score ≥ 36 indicates the presence of fatigue

*ALSFRS-R*, Amyotrophic Lateral Sclerosis Functional Rating Scale- Revised; *FSS*, Fatigue Severity Scale; *CIS*, Checklist Individual Strength, *ALSSQol*, ALS Specific Quality of Life-Revised

**Table 2:** Quality assessment of the included studies using the JBI critical appraisal checklist for prevalence studies

Study ID	Was the sample frame appropriate to address the target population?	Were study participants recruited in an appropriate way?	Was the sample size adequate?	Were the study subjects and the setting described in detail?	Was the data analysis conducted with sufficient coverage of the identified sample?	Were valid methods used for the identification of the condition?	Was the condition measured in a standard, reliable way for all participants?	Was there appropriate statistical analysis?	Was the response rate adequate?
Alencar 2022 a	Yes	Not clear	No	Yes	NA	Yes	Yes	Yes	NA
Alencar 2022 b	Yes	Yes	No	Yes	NA	Yes	Yes	Yes	NA
An 2022 a	Yes	Yes	No	Yes	NA	Yes	Yes	Yes	NA
An 2022 b	Yes	Not clear	No	Yes	NA	Yes	Yes	Yes	NA
Lococo 2012	Yes	Not clear	No	Yes	NA	Yes	Yes	Yes	NA
McElhiney 2009	Yes	Yes	No	Yes	NA	Yes	Yes	Yes	NA
Panitz 2015	Yes	Not clear	No	Yes	NA	Yes	Yes	Yes	NA
Raheja 2016	Yes	Not clear	No	Not clear	NA	Yes	Yes	Yes	NA
Sandstedt 2016	Yes	Not clear	No	Not clear	NA	Yes	Yes	Yes	NA
Vangroenestijn 2017	Yes	Not clear	No	Yes	NA	Yes	Yes	Yes	NA
Vogt 2020	Yes	Not clear	No	Yes	NA	Yes	Yes	Yes	NA

*JBI*, Joanna Briggs Institute; *NA*, Not applicable

### Frequency of fatigue in ALS patients

**Fig. 2:**
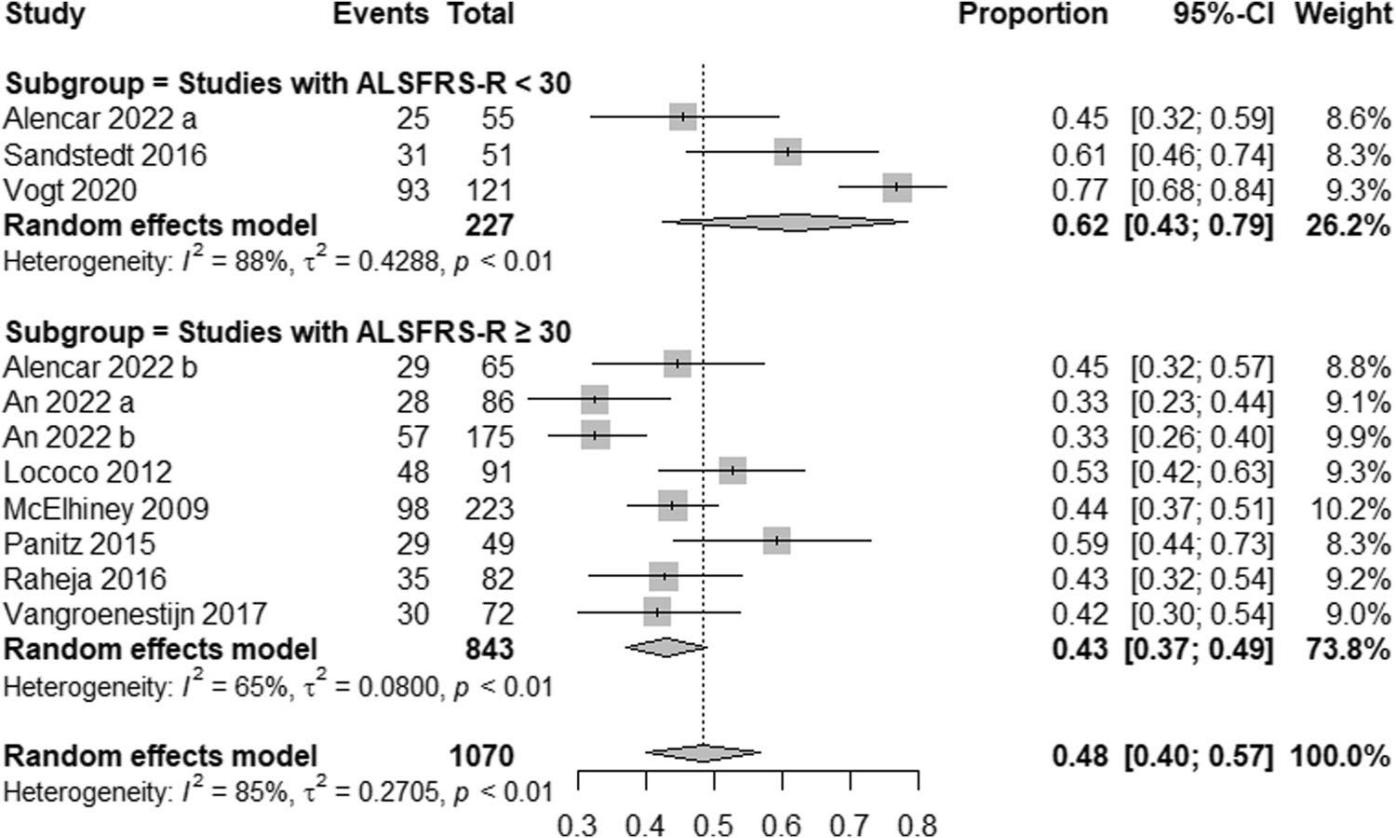
Frequency of fatigue in amyotrophic lateral sclerosis

**Fig. 3:**
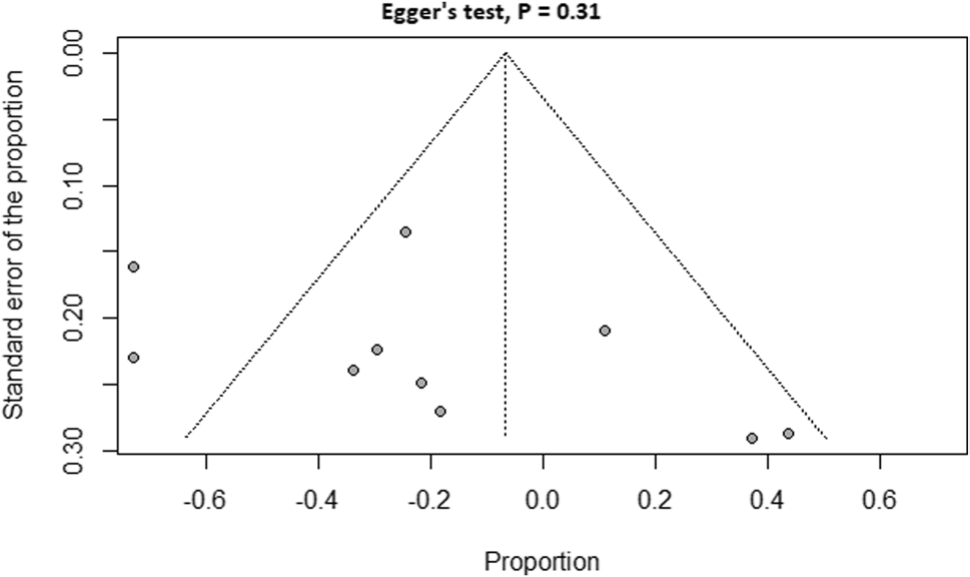
Funnel plot with Egger's test for publication bias

The studies included in the analysis reported a wide range of fatigue prevalence, with values ranging from 32.6% to 76.7%. The studies also showed a significant heterogeneity (*I*^*2*^ = 85%, *p* < 0.01). The pooled prevalence across all studies was 48% (95% confidence interval (CI) = 40% to 57%) as shown in Fig. 2. Our subgroup analysis based on ALSFRS-R showed that the prevalence of fatigue was higher in studies with lower ALSFRS-R scores (< 30) compared to studies with higher ALSFRS-R scores (≥ 30), with a pooled prevalence of 62% (95% CI = 43% to 79%) and 43% (95% CI = 37% to 49%), respectively (Fig. 2**)**. On the other hand, the subgroup analysis based on the tool used to measure fatigue did not reveal significant differences in the prevalence of fatigue between studies that used the FSS and those that utilized the CIS. The pooled prevalence of fatigue was 49% (95% CI = 38% to 59%) for FSS and 51% (95% CI = 33% to 69%) for CIS. The funnel plot was acceptably symmetrical, and Egger’s test was not significant (*P* = 0.31), suggesting a low risk of publication bias (Fig. 3).

One of the studies included in the analysis used both the FSS and CIS tools, with the FSS data being used in the analysis [41]. However, a sensitivity analysis using the CIS data showed that the pooled frequency of fatigue was 47% (95% CI = 39% to 55%) and other analyses did not differ significantly.

### Fatigue correlates in ALS patients

**Fig. 4:**
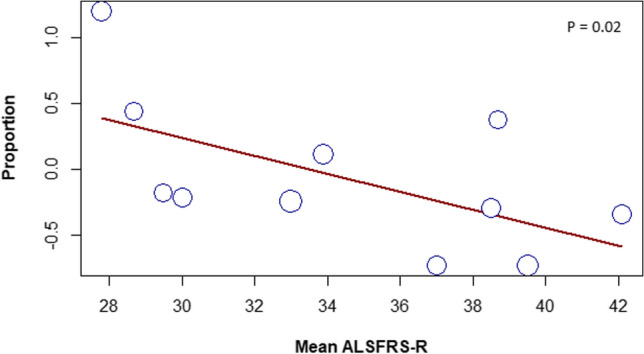
Random-effects meta-regression chart of the association between the frequency of fatigue and ALSFRS-R mean. Every circle represents a study, and the size of the circle represents the weight of the study in the analysis

As shown in Table 3, fatigue was associated with lower functional status and poorer quality of life (QoL) and well-being of patients with ALS across the included studies. Four studies reported a negative correlation between fatigue and ALSFRS-R [9, 37, 40, 41], while two studies found a positive correlation between fatigue and depression [9, 40]. Furthermore, our meta-regression confirmed the negative association between fatigue and ALSFRS-R mean (*P* = 0.02), as shown in Fig. 4. However, no significant associations were found between fatigue and sample size, sex (male number), time from disease onset, and year of publication.

**Table 3:** Reported fatigue associations in the included studies

Study ID	Associations
Alencar 2022 a [7]	Non-functional ambulatory patients had a higher level of fatigue (*P* = 0.026). There was a positive association between fatigue and the inability to walk (*P* = 0.034)
Alencar 2022 b [37]	There was a positive association between fatigue and pain intensity (*P* = 0.001). Fatigue was negatively associated with ALSFRS-R (*P* = 0.003), muscle strength (*P* = 0.004), and quality of life (*P* = 0.001)
An 2022 a [38]	There was a positive association between fatigue and the presence of pain (*P* = 0.032)
An 2022 b [39]	The ALS severity, sleepiness, and daytime dysfunction were associated with a higher risk of fatigue (*P* = 0.002, 0.045, and 0.001, respectively)
Lococo 2012 [9]	Fatigue was negatively associated with ALSFRS-R, forced vital capacity (*P* = 0.001), and sleep quality (*P* = 0.01). Fatigue was positively associated with sleepiness (*P* = 0.007) and depression (*P* = 0.003). Patients with fatigue were significantly more disabled (*P* < 0.001) and more frequently reported difficulties staying asleep (*P* = 0.009) and nocturnal complaints (*P* = 0.002)
McElhiney 2009 [40]	Fatigue was significantly and negatively associated with ALSFRS-R and positively with depression and ALS severity (*P* < 0.001)
Panitz 2015 [41]	Patients with fatigue had lower ALSFRS-R values (*P* < 0.01)
Sandstedt 2016 [43]	Fatigue was associated with worsening health-related quality of life (*P* = 0.01)
Vangroenestijn 2017 [44]	Fatigue was positively associated with participation restrictions (*P* ≤ 0.01)
Vogt 2020 [36]	Fatigue was significantly associated with worse health-related quality of life

*ALSFRS-R*, Amyotrophic Lateral Sclerosis Functional Rating Scale- Revised

## Discussion

### Main findings

Our study aimed to analyze the prevalence and associations of fatigue in ALS patients. Our meta-analysis revealed that almost half of ALS patients (48%) experience clinical fatigue. Additionally, we found that fatigue is significantly associated with poorer functional status in ALS patients, especially on the ALSFRS-R score.

### Interpretations

Amyotrophic lateral sclerosis (ALS) is a neurodegenerative disorder with an unknown origin which leads to the progressive death of upper and lower motor neurons, leading to respiratory failure and death within two to four years from the onset of symptoms [1]. Patients with ALS often experience non motor symptoms, showing a decline in their QoL [45]. Fatigue is one of the common non-motor symptoms in ALS. Fatigue refers to a sensation of exhaustion, weariness, or a decline in physical or mental energy [46]. It is a subjective feeling of being tired and lacking the motivation to continue with physical or mental tasks [46]. In our study, we did not include studies that relied solely on patient complaints or neurophysiological measures [47]. Instead, we focused on these studies that used validated questionnaire, such as FSS, CIS, and ALSSQoL, to accurately measure frequency and severity of fatigue in ALS [47–50]. However, while these questionnaires are effective in measuring the severity of fatigue, they may not explicitly identify fatigue to the respondents. For example, FSS and ALSSQoL questionnaires ask patients to rate the degree of their fatigue, which introduces high subjectivity and the potential for motor weakness to be misinterpreted as fatigue [48, 50]. On the other hand, CIS questionnaire do not contain the term “fatigue” itself, and provides more specific, simple, and less subjective terms, aiming to identify the presence and severity of fatigue [49].

Overall, our findings demonstrate that fatigue is a prevalent issue among patients with ALS, contributing to emotional distress and a lower QoL. Various medical conditions and psychological factors, such as respiratory problems, medication, malnutrition, and depression, may contribute to fatigue in ALS [11]. In our analysis, the pooled prevalence across all included studies was 48%. This result was in line with most of the included studies, as five of them reported a prevalence between 40 to 50% [7, 37, 40–42, 44]. This finding is also consistent with the prevalence of fatigue in other neurological diseases. Siciliano et al., in their meta-analysis, reported a fatigue prevalence of 50% in Parkinson's disease patients [13]. Similarly, Cumming and Alghamdi found that approximately 50% of stroke patients experience fatigue [51, 52]. Furthermore, our analysis revealed a significant association between lower ALSFRS-R scores and fatigue in ALS patients. This association may be attributed to less functionality, lower QoL, higher levels of pain, more advanced disease progression, and more pronounced muscle weakness, as discussed in the study conducted by Alencar et al. [37].

### Strengths and limitations

This study represents the first systematic review and meta-analysis investigating the prevalence of fatigue in patients with ALS. We followed strict inclusion criteria to enhance the accuracy of our results, which may have limited the number of included studies. Our analysis incorporated data from 11 peer-reviewed studies with definitive diagnostic criteria to identify ALS patients. However, there were several limitations to our study. First, the included studies showed significant heterogeneity, which may affect the reliability of the pooled estimates. Different tools were used to measure fatigue across the studies, and most of these tools provide a high degree of subjectivity in identifying fatigue, which may have introduced variability in the results and hindered comparability. Most of the included studies had a cross-sectional design, which limits the ability to establish causality between fatigue and functional status or other variables. Finally, the pooled sample size was relatively small which may limit the generalizability of the results.

### Clinical implications and recommendations

According to our findings, fatigue is a common complaint among people with ALS. It was connected to the degree of severity of the disease as measured by the ALSFRS-R. Fatigue may affect the progression of ALS in multiple ways, as it can result in the loss of movement and a diminished ability to perform daily activities. Moreover, the presence of fatigue can have a substantial impact on the overall well-being and QoL experienced by those diagnosed with ALS. The potential consequences include a decline in engagement with social and recreational activities, heightened reliance on care givers, and a diminished general state of psychological and emotional health [53, 54]. Fatigue has the potential to exacerbate psychological suffering, leading to the experience of various negative emotions such as frustration, despair, and anxiety. Managing the physical constraints associated with ALS in conjunction with the additional burdens posed by fatigue-induced difficulties can have a profound impact on one's mental well-being [11]. Keeping up a healthy diet is essential for people with ALS to preserve their weight and muscle mass, although fatigue can make it difficult to do so [54]. Fatigue, as a common symptom, can affect an individual's ability to adhere to treatment plans, including medication schedules, therapy sessions, and medical appointments [55]. Thus, early intervention for treating fatigue in ALS patients may slow the disease progression and improves the quality of life for the patients [11].

## Conclusions

Our systematic review and meta-analysis found that fatigue is a prevalent and clinically significant symptom in ALS patients. Almost half of ALS patients experience fatigue, highlighting the need for greater attention to non-motor symptoms in ALS patients. Our study also showed significant associations between fatigue and lower functional status and poorer quality of life, confirming the importance of assessing and managing fatigue in ALS patients to improve their overall well-being. Clinicians should include fatigue assessment and management into routine care for ALS patients. Further research is needed to confirm our findings and identify effective interventions for managing fatigue in ALS.
